# The Effects of Chatbot Service Recovery With Emotion Words on Customer Satisfaction, Repurchase Intention, and Positive Word-Of-Mouth

**DOI:** 10.3389/fpsyg.2022.922503

**Published:** 2022-05-31

**Authors:** Jeewoo Yun, Jungkun Park

**Affiliations:** School of Business, Hanyang University, Seoul, South Korea

**Keywords:** chatbot, service quality, emotion words, human chatbot, artificial intelligence, customer satisfaction, repurchase intention, positive word-of-mouth

## Abstract

This study sought to examine the effect of the quality of chatbot services on customer satisfaction, repurchase intention, and positive word-of-mouth by comparing two groups, namely chatbots with and without emotion words. An online survey was conducted for 2 weeks in May 2021. A total of 380 responses were collected and analyzed using structural equation modeling to test the hypothesis. The theoretical basis of the study was the SERVQUAL theory, which is widely used in measuring and managing service quality in various industries. The results showed that the assurance and reliability of chatbots positively impact customer satisfaction for both groups. However, empathy and interactivity positively affect customer satisfaction only for chatbots with emotion words. Responsiveness did not have an impact on customer satisfaction for both groups. Customer satisfaction positively impacts repurchase intention and positive word-of-mouth for both groups. The findings of this study can serve as a priori research to empirically prove the effectiveness of chatbots with emotion words.

## Introduction

Rapidly improving digital technologies have changed the nature of services, customer experiences, and their relationships with companies (Van Doorn et al., 2017). Technologies based on artificial intelligence (AI) are considered a game-changer in many industries (Pillai and Sivathanu, 2020), and the interface between businesses and customers are becoming increasingly technology-driven rather than human-driven (Larivière et al., 2017). Innovative technologies, such as chatbots, AI, and robotics, are disrupting the customer management systems of industries (Bowen and Morosan, 2018; Tussyadiah, 2020). In recent years, the burgeoning reliance on chatbots has culminated in technological improvement (Huang and Rust, 2018). The COVID-19 pandemic has accelerated the use of chatbots in many industries, which, in turn, has encouraged customers to utilize online platforms. Under these circumstances, chatbots constitute a prominent AI system. They are automated programs that offer support and assistance to humans in making purchases and seeking information by communicating through text (Przegalinska et al., 2019; Ashfaq et al., 2020). Chatbots were originally designed to perform simple tasks that require communication through text. However, today, chatbots can also perform complex tasks such as providing shopping recommendations, setting up pre-orders, and performing tasks using location-based services (Araujo, 2018), thus increasing users' accessibility, convenience, and cost-savings (Jang et al., 2021). Chatbots are widely used in various industries, such as finance, tourism, education, and healthcare. Several brands have also adopted the digital service trend by offering 24 x 7 customer support *via* chatbots. As they sell both brand value and image in addition to their products, high-quality services and close relationships with their customers are very important. Chatbots offer a new layer of support, facilitating the accomplishment of service-quality dimensions through personalized services in order to meet customers' needs anytime and anywhere. They are designed to promote future brands and customer relationships by providing information on global offline stores, access to personal-service agents for product care, and conversational interfaces that showcase the craftsmanship behind the products (Chung et al., 2020). However, it has been repeatedly argued that the robotic nature of chatbots (emotionless and artificial interaction) disrupts the close relationship between brands and customers online (Go and Sundar, 2019). Many customers consider chatbots inhuman (Shumanov and Johnson, 2021), and they question their reliability (Rese et al., 2020; Li et al., 2021). They believe that chatbots are clumsier than humans with respect to emotional tasks (Madhavan et al., 2006), and they tend to prefer human-like chatbots (Wexelblat, 1998). Thomas et al. (2018) argued that the conversation style of chatbots in an anthropomorphic context impresses customers. Some researchers have insisted on incorporating warmth in chatbot conversations in order to increase the degree of personification, and the expression of empathy is preferred over emotionless advice (Liu and Sundar, 2018; Roy and Naidoo, 2021). Human-like chatbots that recognize, understand, and express a variety of emotions can contribute toward improving customer impressions and attitudes, particularly toward the service and the company as a whole. This study examines how the effect of the quality of chatbot services on customer satisfaction, repurchase intention, and positive word-of-mouth (WOM) differs when emotion words such as happy, sorry, like, favorite, thank you etc., are introduced in the communication systems of brands. Numerous studies have verified the relationship between service quality and customer satisfaction, WOM, and repurchase intention. However, few have investigated the service quality with a focus on chatbots, particularly the difference between the effects of emotional and unemotional conversations.

## Literature Review

### Chatbot Service

A chatbot “is a machine conversational system that interacts with human users using natural conversational language” (Shawar and Atwell, 2005, p. 489) or “an artificial construct designed to converse with human beings using natural language as input and output” (Brennan, 2006, p. 61). Lester et al. (2004) define chatbots as technologies that engage users in text-based or task-oriented conversations using natural language on websites and applications. Originally created for entertainment purposes, they used simple techniques of matching keywords (Shawar and Atwell, 2007). However, advances in disciplines such as natural language processing and AI have substantially enhanced the capabilities of modern chatbots in textual and spoken communication (Shah et al., 2016). Firms from various industries have utilized these functions and employed chatbots for client interactions (Følstad and Brandtzæg, 2017). Winkler and Soellner (2018) described four advantages of chatbots: replacement of a personal assistant, facilitation of real-time interactions, prediction of customer questions, and sophisticated problem analysis. Whereas, human employees require time and effort to understand and learn service processes, chatbots are devoid of human error and weariness and work consistently, providing homogeneous services with high degrees of reliability (Wirtz et al., 2018; Meyer-Waarden et al., 2020). Therefore, chatbot can be defined as around-the-clock personal assistants that help build important customer–brand relationships. Chatbot technology adoption is a new area of research that is being examined from several perspectives. First, the technical aspects of chatbots have been investigated, such as speech conversation system technologies (Abdul-Kader and Woods, 2015) and programming methodologies (Long et al., 2019). Second, several studies have focused on human and chatbot interactions to enhance customer purchases (Luo et al., 2019) and the willingness of users to communicate with chatbots (Mirnig et al., 2017). Third, studies have examined the utilization of chatbot technologies in customer service in order to assess their usability (Kang and Kim, 2017) and impact on customer satisfaction (Chung et al., 2020) in various industries such as finance, tourism, education, and healthcare (Quah and Chua, 2019; Gunawan et al., 2020; Zhang et al., 2020; Yin et al., 2021). According to Følstad and Brandtzæg (2017), major companies like Google, Facebook, and Microsoft consider chatbots as the “next big thing.” Chatbot optimize the customers' time by providing easy access to products and provide in-depth insights on product performance (Zhang et al., 2019). Chung et al. (2020) reported that chatbots increase brand satisfaction by engaging customers to provide interactive assistance. Therefore, many brands have incorporated chatbots, recognizing their bright prospects and increasing popularity (Lee and Choi, 2017). However, despite the increasing use of chatbots by brands, related studies are significantly fewer than those for other industries. There have been few attempts to verify the important quality dimensions of chatbot services, particularly for brands, which underscores the importance of this study.

### Theoretical Background (SERVQUAL)

In the second half of the twentieth century, several researchers attempted to develop systems for measuring the quality of services (Parasuraman et al., 1985). Early literature has provided a wide range of definitions for service quality. One perspective has recognized technical quality to be measured as what the customer actually receives from the service and functional quality as the manner of service delivery (Grönroos, 1984). A second perspective has indicated that services are jointly introduced from providers to recipients over three dimensions: physical features, corporate image or reputation, and interaction between first-line service providers and end customers (Lehtinen and Lehtinen, 1991). After multiple refinements, the SERVQUAL theory centered on five dimensions: reliability, tangibility, responsiveness, empathy, and assurance (Parasuraman et al., 1988). SERVQUAL has been developed further and has become a key tool in measuring the quality of services. The developments in SERVQUAL include E-SERVQUAL (Parasuraman et al., 2005), the hierarchical model, and SERVPERF (Cronin and Taylor, 1992, 1994). SERVQUAL has been used in many industries and has remained the most common instrument for assessing service quality in research and practical fields. Asubonteng et al. (1996), Seth et al. (2005), and Ladhari (2009) among others, consider this model a valuable tool in assessing customer satisfaction. Many research efforts have investigated the relationship between quality of services and customer satisfaction (Zeithaml et al., 1996; Olorunniwo et al., 2006; Kitapci et al., 2013). Several studies have indicated that a positive relationship exists between perceived service quality and customer satisfaction, or service quality precedes customer satisfaction (Lee et al., 2000; Tam, 2004; Pan et al., 2010). Moreover, high service quality elevates the brand name and increases brands' excellence in service delivery (Parasuraman et al., 1988). SERVQUAL is a well-established tool for benchmarking as it undergoes significant field-testing and improvement (Dagger et al., 2007). The SERVQUAL model developed by Parasuraman et al. (1985) is chosen here because it is the most widely employed model in managing and measuring the quality of services in various industries. However, tangibility, including physical facilities, personnel appearance, and equipment, does not apply to the chatbot service context. Tangibility refers to the importance of the physical environment that influences customers' behaviors (Zeithaml et al., 1990). Parasuraman et al. (1988, 1991) interpreted the ambient conditions, such as the atmosphere, temperature, noise, and smell of a store, as tangible dimensions of service quality, as they can be directly perceived by human senses. Since such ambient conditions do not pertain to chatbots, it is reasonable not to involve tangibility in chatbot conversations. Customers expect to have the same levels of interpersonal interactions online as they do offline (Sivaramakrishnan et al., 2007). Satisfying customers' expectations for interactions with service agents can result in the satisfaction of customers, positive WOM, loyalty, intentions of favorable purchase, and increased profits (Reynolds and Beatty, 1999). Go and Sundar (2019) assume that interactivity is essential for improving the humanity of chatbot-based systems. The human-like characteristics of chatbots improve the quality of conversations and promote emotional and social connections (Biocca et al., 2003; Bente et al., 2008). Moreover, the enhanced psychological effect of interacting with a chatbot may lead to a good attitude toward the website or brand (Araujo, 2018; Go and Sundar, 2019). Consequently, customers are influenced by online interactions that are similar to real-world ones in terms of purchase decisions and advice, time savings, and/or para-social advantages (Holzwarth et al., 2006). The interactivity of chatbots is important for achieving high-quality customer services. However, it has not been considered in many studies. Considering the interactivity dimension instead of a tangible one, this study examines the conceptual model of the improved SERVQUAL theory, which includes reliability, assurance, responsiveness, interactivity, and empathy.

## Hypothesis Development

### Reliability of Chatbot Services

The reliability of organizations indicates their ability to deliver the promised service accurately and dependably while ensuring the safety of personal information (Parasuraman et al., 1988; Janda et al., 2002). Many researchers have considered reliability to be the most important indicator of the quality of service (Dhingra et al., 2020). Wolfinbarger and Gilly (2003) argue that organizational reliability highly influences customers' judgments on service quality online. According to Zhu et al. (2002), online systems' reliability positively impacts customers' satisfaction and their perceived quality of the overall service. Lee and Lin (2005) strongly believed that reliability can significantly predict the overall quality of services, purchase intentions, and customer satisfaction. Moreover, they emphasized the importance of reliability in technology-based services. Accordingly, we propose the following hypothesis:

H1: The reliability of chatbot services positively impacts customer satisfaction with the services.

### Responsiveness of Chatbot Services

Responsiveness is a traditional SERVQUAL dimension and represents the organization's willingness and ability to deliver prompt services and reactions in case customers have inquiries or problems (Zeithaml, 2002). The organization's ability to respond timely to complaints and order confirmations through email has been considered an important aspect of customers' online evaluations (Sharma, 2018). This is because customers expect prompt online responses to their inquiries from the organization (Liao and Cheung, 2002). Responsiveness plays a central role in communicating with customers and can support internet-based service providers in implementing various service functions on the website (Lee and Kozar, 2006). In an online environment, organizations must be courteous in their customer service, and they should provide an adequate response to the customer. The responsiveness of chatbots is an essential quality attribute that can significantly improve the performance of chatbot systems (Li et al., 2021). Thus, we propose the following hypothesis:

H2: The responsiveness of chatbot services positively impacts customer satisfaction with the services.

### Assurance of Chatbot Services

Parasuraman et al. (1988) defined assurance as the knowledge and courtesy of an employee, and the ability to inspire trust and confidence. Research on the shopping industry has shown that employees' language skills, attitudes, efficiency (Heung and Cheng, 2000), and knowledge of the sales staff (Lin and Lin, 2006) are given significant importance in determining customer satisfaction. Assurance, measured by security and trustworthiness in e-commerce settings, has also been supported as an independent variable with a positive relationship with customer satisfaction (Ribbink et al., 2004; Kassim and Abdullah, 2010). Li et al. (2021) found that assistance has a significant relationship with confirmation and a positive relationship with satisfaction. Assurance refers to trust, a feeling of safety, as well as a sense of comfort in conversations with and knowledge of the chatbot. Based on these discussions, we propose the following hypothesis:

H3: The assurance of chatbot services positively impacts customer satisfaction with the services.

### Interactivity of Chatbot Services

According to Heeter (1989), interactivity is defined as the extent of similarity between technology and human discourse in the communication exchange. Neuhofer et al. (2015) opine that interactivity is occasionally considered a pivotal element in providing customers with personalized services and ultimately increasing customer engagement. A study on e-tailing indicates that perceived interactivity positively impacts customers' pleasant feelings in their e-commerce experiences (Yoo et al., 2010). Moreover, Shin et al. (2013) and Cho et al. (2019) found that smart products' perceived interactivity helps in creating positive feelings and satisfaction with the product. As chatbot services are smart services, it can be estimated that a high level of interaction positively impacts customer satisfaction. Godey et al. (2016) believe that interactivity positively impacts customer-brand relationships in luxury businesses. Thus, we propose the following hypothesis:

H4: The interactivity of chatbot services positively impacts customer satisfaction with the services.

### Empathy of Chatbot Services

Murray et al. (2019) defined empathy as the ability to understand, identify, and respond to people's thoughts, behaviors, feelings, and experiences. Accordingly, empathy is a multidimensional construct that involves affective, cognitive, and compassionate perceptions (Powell and Roberts, 2017). Scholars have argued that in the traditional service setting, customers will be more satisfied with a brand when employees espouse empathetic attitudes (Markovic et al., 2018). Moreover, Lee et al. (2011) concluded that employee empathy directly impacts customers' positive emotions, and there is a significant positive association between positive emotions and satisfaction with the employee relationship. The empathetic ability of social robots significantly affects the intention to use robots (de Kervenoael et al., 2020). Research has examined consumers' responses to text-based chatbots in the e-commerce context. It has shown that consumers prefer chatbots that can understand their needs and respond to them, ultimately yielding positive perceptions of chatbots having high empathy (Chung et al., 2020). Thus, we propose the following hypothesis:

H5: The empathy of chatbot services positively impacts customer satisfaction with the services.

### Customer Satisfaction With Chatbot Services, Repurchase Intention, and Positive WOM

Customer satisfaction represents the difference between customers' expectations from services and products before purchase and their perceived service quality after purchase (Oliver, 1980). It is the combined output of customers' perceptions, evaluations, and psychological reactions to their experience of consuming a product or service (George and Kumar, 2014). Thus, customer satisfaction is widely acknowledged as a critical component of marketing success that has a vital role in enhancing the competitiveness of firms (Kant and Jaiswal, 2017).

According to Bayraktar et al. (2012), repurchase intention is defined as a personal judgment of availing a service more than once and deciding to participate in a future activity with the same service provider in the same form. Customer satisfaction usually precedes a repurchase intention. Liao et al. (2017) found a significant impact of consumer satisfaction on repurchase intention in the service domain, and Larivière et al. (2016) argued that customer satisfaction increases the profitability of the service provider by fostering customers' repurchase intentions.

WOM is a behavior on part of consumers, wherein they inform others about their experiences with particular products and services (Bowman and Narayandas, 2001). This can provide a significant competitive advantage and have a strong impact on product and service perception (Dagger et al., 2007). Nguyen and Romaniuk (2014) found that WOM has a greater impact than general advertising on individuals. Akinci and Aksoy (2019) found that customer satisfaction plays an important role in WOM. Verkijika and De Wet (2019) argued that users communicate positively through WOM if they are satisfied with their initial usage experience.

Many scholars have demonstrated that satisfaction is an antecedent with a significant effect on repurchase intention and WOM in various industries (Kassim and Abdullah, 2010; Kitapci et al., 2014; Meilatinova, 2021). Thus, we propose the following hypotheses:

H6: Customer satisfaction with chatbot services positively impacts repurchase intention.
H7: Customer satisfaction with chatbot services positively impacts positive word-of-mouth.

**Figure 1:**
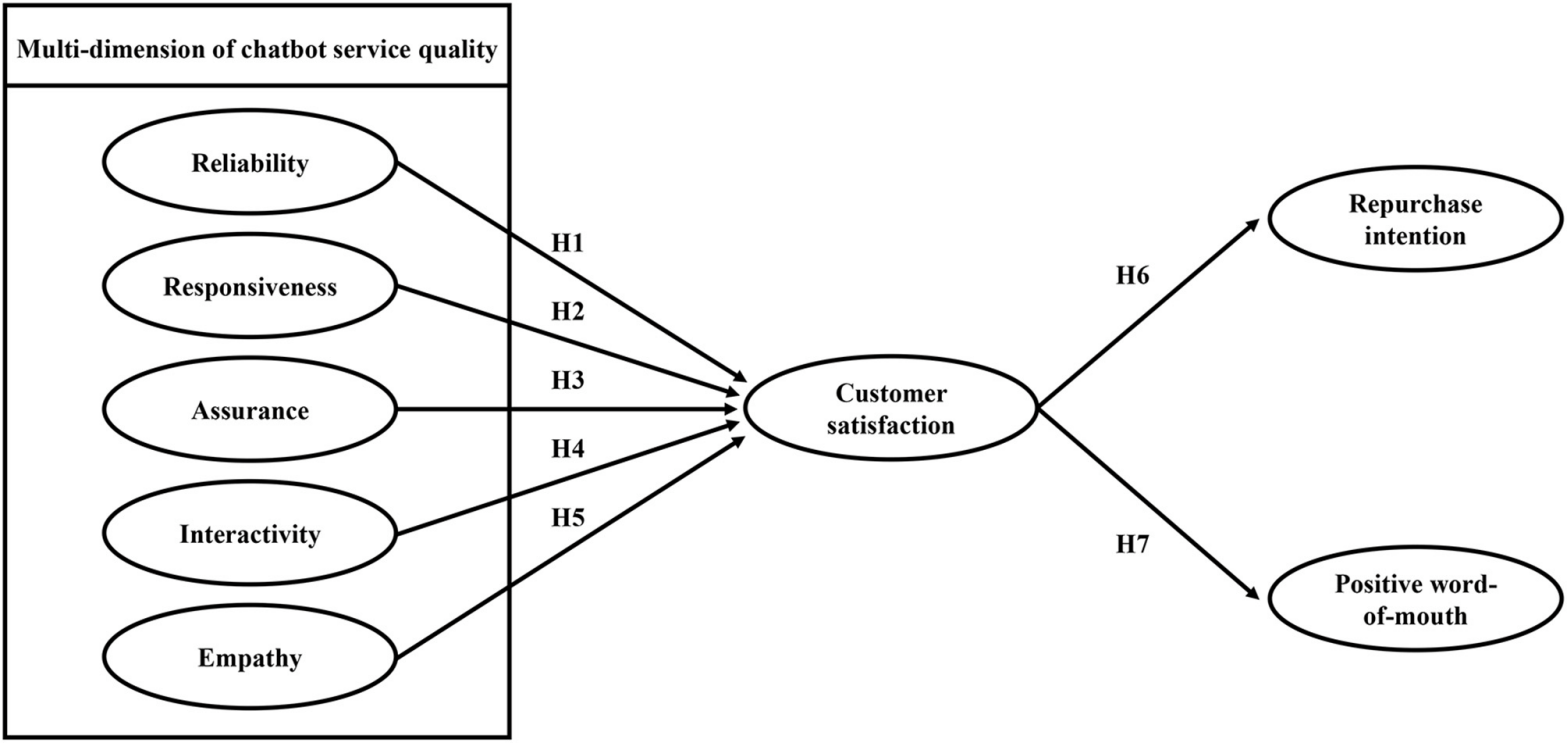
Conceptual framework of the multi-dimension of chatbot service quality. This conceptual framework is an improved version of Parasuraman's (1988) SERVQUAL model considering the interactivity dimension to fit the chatbot service.

Figure 1 presents the conceptual framework of the perceived quality of chatbot services.

## Methodology

### Research Design

This study was designed with due consideration for two scenarios (a chatbot with emotion words vs. a chatbot without emotion words), and a lab test was conducted. A service failure scenario was used to investigate the service recovery quality of the chatbot in such a situation. The respondents were selected from among people experienced in purchasing products from online brand shops. They were directed to order goods from their favorite brands. However, a service failure occurred with their orders, which was either a delivery problem (late delivery or wrong address) or poor product quality (wrong product/size/color or a broken/scratched product). The respondents visited the official website of the brand to report their issues, and an automatic chatbot appeared as a representative customer service agent to solve their problems. The respondents were randomly assigned to one of two simulated situations (a chatbot with emotion words vs. a chatbot without emotion words). They were invited to experience a simulated conversation with a chatbot designed by a group of Ph.D. students. A set of emotion words generated for a chatbot was selected from Huo et al. (2020), which included words like “sorry,” “like,” “truly,” “thank you,” and “pity” (Supplementary Table 1).

### Sample Characteristics

Data were collected over a period of 2 weeks (May 2021). The ratio of the total number of samples was derived by adding the ratio of respondents in the two situations and halving it. Among the 380 respondents, 56.3% were male, and 43.7% were female. Those aged between 20 and 29 years (43.4%), and 30 and 39 years (40.3%) accounted for the largest portions of the sample. Only 1.05% of the respondents were aged over 60 years. Most respondents (28.9%) earned between USD 4,501 and USD 6,000 monthly. Those who earned between USD 1,500 and USD 3,000 ranked second (28.7%), and those earning between USD 3,001 and USD 4,500 ranked third (17.4%). Most respondents had bachelor's degrees from a college/university (68.7%), followed by master's (28.2%) and high school (2.9%) degrees (Table 1).

**Table 1:** Demographic characteristics of the respondents.

**Characteristics**		**Emotion (*****n*** **= 192)**		**No emotion (*****n*** **= 188)**	
		**Frequency**	**Percentage**	**Frequency**	**Percentage**
Gender	Male	105	54.7	109	58.0
	Female	87	45.3	79	42.0
Age	20–29 years	78	40.6	87	46.3
	30–39 years	76	39.6	77	41.0
	40–49 years	35	18.2	13	6.9
	50–59 years	1	0.5	9	4.8
	60 years or older	2	1.0	2	1.1
Monthly income	< USD 1,500	30	15.6	22	11.7
	USD 1,500–USD 3,000	48	25	61	32.4
	USD 3,001–USD 4,500	38	19.8	28	14.9
	USD 4,501–USD 6,000	59	30.7	51	27.1
	Over USD 6,000	17	8.9	26	13.8
Education level	Less than High school	0	0	0	0
	High school	3	1.6	8	4.3
	College/University	141	73.4	120	63.8
	Master's degree	47	24.5	60	31.9
	Doctorate/PhD	1	0.5	0	0
	Others	0	0	0	0
Marital status	Single	61	31.8	67	35.6
	Married	131	68.2	120	63.8
	Divorced	0	0	1	0.5

### Development of the Measurement Model

To measure the service quality of chatbots, five dimensions, namely interactivity, reliability, responsiveness, assurance, and empathy with 15 items were developed by drawing from Parasuraman et al. (1988) and Li et al. (2021). Six items were adopted from Parasuraman et al. (1988), Li et al. (2021), and Bagherzadeh et al. (2020) to measure customer satisfaction and positive WOM. The dimensions were measured using a seven-point Likert scale (1 = strongly disagree, 2 = disagree, 3 = slightly disagree, 4 = neutral, 5 = slightly agree, 6 = agree, 7 = strongly agree). Repurchase intention was measured using three items on a semantic scale that ranged from 1 to 7 (improbable to very probable, impossible to possible, no chance to certain), which was a modified version of the scale in Moriuchi et al. (2021). A total of 24 items were extracted from 8 dimensions and used in the final measurement (Supplementary Table 2).

## Results

### Measurement Model

The analysis was performed through SPSS 26.0 and AMOS 22.0. Exploratory factor analysis, confirmatory component analysis, correlation tests, and reliability tests were used to examine the measurement's internal consistency and validity. Subsequently, a structural equation model was constructed to test the hypotheses proposed in this study. To test the dimensionality of the perceived service-quality dimensions, all 15 items were analyzed using Varimax rotation through exploratory factor analysis. The criterion of meaningful factor loading was set to 0.4 (Table 2).

**Table 2:** Results of exploratory factor analysis.

	**ASS**	**INT**	**RES**	**EMP**	**REL**
**Component (Emotion)**					
ASS1	**0.902**				
ASS2	**0.892**				
ASS3	**0.868**				
INT3		**0.898**			
INT2		**0.873**			
INT1		**0.856**			
REL1			**0.885**		
REL2			**0.835**		
REL3			**0.820**		
EMP2				**0.877**	
EMP3				**0.846**	
EMP1				**0.822**	
RES3					**0.872**
RES2					**0.858**
RES1					**0.807**
**Component (No emotion)**					
ASS1	**0.854**				
ASS2	**0.846**				
ASS3	**0.832**				
INT1		**0.874**			
INT2		**0.861**			
INT3		**0.855**			
REL2			**0.892**		
REL1			**0.862**		
REL3			**0.850**		
EMP3				**0.873**	
EMP2				**0.872**	
EMP1				**0.825**	
RES2					**0.850**
RES3					**0.846**
RES1					**0.838**

*ASS, assurance; INT, interactivity; REL, reliability; EMP, empathy; RES, responsiveness*.

The assessment of a variety of goodness-of-fit measures to evaluate the overall model fit produced the following results (chatbot with emotion words: CMIN/DF = 1.280, GFI = 0.890, IFI = 0.972, TLI = 0.965, CFI = 0.972, RMSEA = 0.038; chatbot with no emotion words: CMIN/DF = 1.443, GFI = 0.878, IFI: 0.955, TLI: 0.943, CFI: 0.954, RMSEA = 0.049). All the goodness-of-fit indices were within acceptable limits. The measurement model was tested for reliability and convergent validity, which was assessed through the estimate, Cronbach's alpha, construct reliability (CR), and average variance extracted (AVE) (Hair et al., 2013). Reliability demonstrated by Cronbach's alpha and CR value exceeded 0.7, and the AVE of all constructs was above 0.5. Thus, the results indicate good reliability and convergent validity as suggested by previous researchers (Fornell and Larcker, 1981; Hair et al., 2006; Table 3). Table 4 presents the results of the correlations matrix among constructs that have a significant relationship and shows the constructs' mean and standard deviation.

**Table 3:** Reliability and validity tests: with and without emotion words.

**Variable**	**Indicator**	**Estimate**		***t*** **-value**		**AVE**		**Cronbach's** ***a***		**CR**	
		**With**	**W/out**	**With**	**W/out**	**With**	**W/out**	**With**	**W/out**	**With**	**W/out**
Reliability	REL1	0.870	0.825	10.756	12.164	0.642	0.693	0.828	0.867	0.843	0.872
	REL2	0.771	0.848	10.188	12.442						
	REL3	0.758	0.825	–	–						
Responsiveness	RES1	0.732	0.739	9.749	8.516	0.613	0.577	0.820	0.803	0.825	0.804
	RES2	0.760	0.800	10.012	8.621						
	RES3	0.851	0.739	–	–						
Assurance	ASS1	0.904	0.822	13.159	10.992	0.721	0.629	0.885	0.830	0.886	0.835
	ASS2	0.850	0.733	12.662	10.035						
	ASS3	0.790	0.821	–	–						
Interactivity	INT1	0.769	0.883	11.639	11.853	0.677	0.690	0.859	0.868	0.862	0.869
	INT2	0.828	0.834	12.513	11.509						
	INT3	0.868	0.771	–	–						
Empathy	EMP1	0.776	0.786	10.273	10.004	0.630	0.628	0.825	0.822	0.836	0.835
	EMP2	0.805	0.813	10.501	10.150						
	EMP3	0.799	0.778	–	–						
Satisfaction	SAT1	0.801	0.830	–	–	0.685	0.682	0.862	0.854	0.866	0.865
	SAT2	0.776	0.874	11.417	12.842						
	SAT3	0.900	0.771	12.982	11.435						
Repurchase intention	RI1	0.779	0.806	–	–	0.658	0.657	0.828	0.834	0.852	0.852
	RI2	0.807	0.835	11.090	11.471						
	RI3	0.846	0.790	11.472	10.995						
Positive WOM	WOM1	0.960	0.959	–	–	0.569	0.614	0.809	0.833	0.791	0.822
	WOM2	0.582	0.636	9.315	10.538						
	WOM3	0.668	0.720	11.548	13.014						

*With, emotion words; W/out, no emotion words; AVE, average variance extracted; CR, construct reliability*.

*Emotion words: CMIN/DF = 1.280; GFI = 0.890; IFI = 0.972; TLI = 0.965; CFI = 0.972; RMSEA = 0.038*.

*No emotion words: CMIN/DF = 1.443; GFI = 0.878; IFI = 0.955; TLI = 0.943; CFI = 0.954; RMSEA = 0.049*.

**Table 4:** Construct means, standard deviations, and correlations.

	**RI**	**REL**	**INT**	**EMP**	**ASS**	**RES**	**SAT**	**WOM**
**Emotion**								
RI	**0.811**							
REL	0.343	**0.801**						
INT	0.418	0.173	**0.823**					
EMP	0.366	0.306	0.199	**0.793**				
ASS	0.435	0.242	0.190	0.292	**0.849**			
RES	0.336	0.328	0.150	0.192	0.112	**0.783**		
SAT	0.458	0.363	0.464	0.361	0.347	0.194	**0.827**	
WOM	0.421	0.314	0.375	0.343	0.270	0.340	0.532	**0.754**
Means	5.288	5.413	4.469	5.118	5.089	3.807	4.752	5.056
SD	0.903	0.936	1.198	1.136	1.151	1.187	1.134	1.066
**No emotion**								
RI	**0.811**							
REL	0.244	**0.833**						
INT	0.233	0.428	**0.831**					
EMP	0.332	0.109	0.145	**0.792**				
ASS	0.422	0.273	0.357	0.334	**0.793**			
RES	0.088	−0.109	0.034	0.073	0.038	**0.760**		
SAT	0.489	0.386	0.251	0.181	0.365	−0.060	**0.826**	
WOM	0.394	0.141	0.191	0.110	0.402	−0.032	0.430	**0.784**
Means	5.390	5.082	4.943	5.541	4.307	4.676	4.897	5.076
SD	0.895	1.100	1.238	0.868	1.221	1.104	1.121	1.054

*The square roots of the AVE for each construct are presented in bold on the diagonal of the correlation matrix. RI, repurchase intention; REL, reliability; INT, interactivity; EMP, empathy; ASS, assurance; RES, responsiveness; SAT, customer satisfaction; WOM, positive word-of-mouth; SD, standard deviation*.

### Structural Model

To test the hypotheses, we used the structural equation model. The overall fit indices showed an acceptable fit to the data (chatbot with emotion words: CMIN/DF = 1.457; GFI = 0.872; IFI = 0.953; TLI = 0.944; CFI = 0.952; RMSEA = 0.049; chatbot with no emotion words: CMIN/DF = 1.527; GFI = 0.867; IFI = 0.943; TLI = 0.932; CFI = 0.942; RMSEA = 0.053). Chatbot service qualities had partially positive impacts on customer satisfaction. For the chatbot with emotion words, reliability (β = 0.202^*^), assurance (β = 0.194^**^), interactivity (β = 0.375^***^), and empathy (β = 0.186^*^) positively impact customer satisfaction, thereby supporting H1, H3, H4, and H5. Responsiveness (β = 0.062; *P*-value = 0.408) did not have a positive effect on customer satisfaction. For the chatbot with no emotion words, only reliability (β = 0.288^**^) and assurance (β = 0.291^**^) positively impact customer satisfaction, thus supporting H1 and H3. Customer satisfaction positively impacts repurchase intention and positive WOM in both cases, namely with and without emotion words, as shown in Table 5, supporting H6 and H7. Thus, satisfaction is an important premise that impacts customer behavior regardless of the chatbot's humanity (Table 5).

**Table 5:** Results of structural equation modeling.

**Hypothesized paths**	**Emotion**				**No emotion**			
	**β**	**t**	***p***	**Result**	**β**	**t**	***p***	**Result**
H1: Reliability	0.202	2.550	0.011	Supported	0.288	3.244	0.001	Supported
→ Customer satisfaction								
H2: Responsiveness	0.062	0.827	0.408	Not	–	–	0.631	Not
→ Customer satisfaction				Supported				Supported
H3: Assurance	0.194	2.652	0.008	Supported	0.291	3.161	0.002	Supported
→ Customer satisfaction								
H4: Interactivity	0.375	4.996	***	Supported	0.030	0.332	0.740	Not
→ Customer satisfaction								Supported
H5: Empathy	0.186	2.382	0.017	Supported	0.078	0.940	0.347	Not
→ Customer satisfaction								Supported
H6: Customer satisfaction	0.517	6.045	***	Supported	0.521	6.153	***	Supported
→ Repurchase intention								
H7: Customer satisfaction	0.572	7.605	***	Supported	0.456	5.908	***	Supported
→ Positive WOM								

*p < 0.05, **p < 0.01,

*** *p < 0.001*.

*Emotion words: CMIN/DF = 1.457; GFI = 0.872; IFI = 0.953; TLI = 0.944; CFI = 0.952; RMSEA = 0.049*.

*No emotion words: CMIN/DF = 1.527; GFI = 0.867; IFI = 0.943; TLI = 0.932; CFI = 0.942; RMSEA = 0.053*.

## Discussion and Conclusion

Owing to technological advancements, businesses can exploit AI systems such as chatbots, to improve their marketing efforts and maintain continuous customer relationships. However, the problem of chatbots' robotic nature interrupting effective communication with customers has been recently argued, insisting on the adoption of human-robot interactions. To overcome this problem, this study sought to examine how the service quality of chatbots with and without emotion words, as perceived by customers, affects customer satisfaction, repurchase intention, and positive WOM. The key findings are summarized below.

First, the results showed that reliability and assurance positively impact customer satisfaction with and without emotion words in chatbot conversations. This is consistent with Zhu et al. (2002), Lee and Lin (2005), and Kitapci et al. (2014). Lee and Lin (2005) studied online shopping experiences and found that reliability affects customer satisfaction. Kitapci et al. (2014) studied the healthcare industry and estimated that assurance affects customer satisfaction. Zhu et al. (2002) studied the IT-based financial sector and found that reliability and assurance influence customer satisfaction. Reliability can be considered very important, particularly for brands that sell brand image and value, not just products. Prominent brands have successfully maintained their reputation for a long time as their customers trust the quality of their products and believe in their ability to deliver the promised services efficiently. Customers expect their flawless in-store experience to be replicated online. This study confirmed that assurance, including employee knowledge, courtesy, confidence in their ability, and trust, should be considered important in chatbot services. Brands should convince customers that chatbots can complete tasks properly online, where they serve as replacements for live employees.

Second, responsiveness did not affect customer satisfaction in both cases. This shows that customers focus more on accurate and reliable services rather than rapid responses. Alternatively, they may have low expectations of chatbot responsiveness as they may understand that chatbots require time to comprehend the script. However, if brands improve chatbot service systems by supplementing the responsiveness of their chatbots, it will have a significant impact on customer satisfaction.

Third, the empathy and interactivity of chatbots with and without emotion words had different influences on customer satisfaction. Empathy and interactivity had a positive effect on customer satisfaction when chatbots used emotion words but did not affect customer satisfaction when chatbots did not use emotion words. Empathy, which encompasses consideration for customers and personal intimacy, is most important for brands, and studies have claimed that they attempt to empathize and communicate with customers to enhance their satisfaction (Chung et al., 2020). This study determined that chatbots with emotion are more familiar with customers, and that this leads to increased satisfaction. The interactivity of chatbots is important in online communication, where frontline employees are not proximate to the customers. In social impact theory, immediacy or closeness can be a major determinant for increased communication (Sands et al., 2020). Interactivity, which encompasses prompt reactions and problem-solving, can lead to high customer satisfaction and sustain close relationships between customers and the brand.

### Implications

#### Theoretical Implications

This study has the following theoretical implications. First, it extends the theoretical framework of the research on chatbot service quality by adopting the interactivity dimension, which has rarely been investigated in the context of brands. Thus, this adds a new concept to the SERVQUAL model.

Second, this study investigated the emotional factors in chatbot systems by providing new insights to the notion that emotional chatbots can provide customers with a far more effective communication service. This study also verified that chatbots without emotion words can offer only reliability and assurance, whereas chatbots with emotion words can offer interactivity and empathy in addition to the above two factors. This study provides experiential evidence for the effects of emotional chatbot services and contributes to the literature on its application in various industries incorporating AI-based services.

#### Practical Implications

The results also have several important managerial implications. First, the verification of emotional chatbot effects implies that corporate marketing managers must adopt emotional attributes for chatbot services by reducing artificial and mechanical aspects while developing new service domains online. Second, interactivity and empathy for customers has a positive influence on customer satisfaction for emotional chatbot services only. This means that a brand communication strategy based on interactivity and empathy are very important for brands that sell not only products but also brand image and value. This implies that brands must establish interactive communication strategies to maintain their core image in order to secure their unique market positions (Liu et al., 2012). It also implies that smooth and accurate interactions are effective in building a positive brand image (Emmers-Sommer, 2004). Third, this study indicated that the responsiveness of chatbot services is not effective in achieving customer satisfaction with or without the emotional aspect, even though a rapid response is essential to maintain a continuous relationship with customers (Gummerus et al., 2004). This means that the responsiveness of chatbots must be improved to strengthen customer relationships. Thus, corporate technical managers should explore routes to improve the responsiveness of their chatbot services.

### Limitations and Directions for Future Research

As with all empirical research, this study has some limitations, which can be treated as opportunities for further research. First, this study examined the quality of chatbot services provided by brands. Thus, a more detailed investigation on the effect of chatbot services in other service domains is essential for generalizability. Second, this study investigated the differences in service quality between chatbots with and without emotion words in conversations with customers. Future research should include an integrated study comparing the differences between human agents using emotion words and those not comparative study may offer a more meaningful conclusion. Third, as this study verified the effect of emotional language in chatbot services, future research should examine the use of other measures such as voice and facial expressions. Finally, this study surveyed a specific area, that is, the USA, which may limit the universality of the results. Thus, future empirical studies must include other countries and outcome variables for generalization and objective comprehension.

## Data Availability Statement

The raw data supporting the conclusions of this article will be made available by the authors, without undue reservation.

## Author Contributions

All authors listed have made a substantial, direct, and intellectual contribution to the work and approved it for publication.

## Conflict of Interest

The authors declare that the research was conducted in the absence of any commercial or financial relationships that could be construed as a potential conflict of interest.

## Publisher's Note

All claims expressed in this article are solely those of the authors and do not necessarily represent those of their affiliated organizations, or those of the publisher, the editors and the reviewers. Any product that may be evaluated in this article, or claim that may be made by its manufacturer, is not guaranteed or endorsed by the publisher.

## References

[B1] Abdul-Kader S. A. Woods J. C. (2015). Survey on chatbot design techniques in speech conversation systems. Int. J. Adv. Comput. Sci. Appl. 6, 72–80. 10.14569/IJACSA.2015.060712

[B2] Akinci S. Aksoy S. (2019). The impact of service recovery evaluation on word-of-mouth intention: a moderated mediation model of overall satisfaction, household income and gender. Tour. Manage. Perspect. 31, 184–194. 10.1016/j.tmp.2019.05.002

[B3] Araujo T. (2018). Living up to the chatbot hype: the influence of anthropomorphic design cues and communicative agency framing on conversational agent and company perceptions. Comput. Hum. Behav. 85, 183–189. 10.1016/j.chb.2018.03.051

[B4] Ashfaq M. Yun J. Yu S. Loureiro S. M. C. (2020). I, Chatbot: Modeling the determinants of users' satisfaction and continuance intention of AI-powered service agents. Telemat. Inform. 54:101473. 10.1016/j.tele.2020.101473

[B5] Asubonteng P. McCleary K. J. Swan J. E. (1996). SERVQUAL revisited: a critical review of service quality. J. Serv. Mark. 10, 62–81. 10.1108/08876049610148602

[B6] Bagherzadeh R. Rawal M. Wei S. Torres J. L. S. (2020). The journey from customer participation in service failure to co-creation in service recovery. J. Retail. Consum. Serv. 54:102058. 10.1016/j.jretconser.2020.102058

[B7] Bayraktar E. Tatoglu E. Turkyilmaz A. Delen D. Zaim S. (2012). Measuring the efficiency of customer satisfaction and loyalty for mobile phone brands with DEA. Expert Syst. Appl. 39, 99–106. 10.1016/j.eswa.2011.06.04114619154

[B8] Bente G. Rüggenberg S. Krämer N. C. Eschenburg F. (2008). Avatar-mediated networking: increasing social presence and interpersonal trust in net-based collaborations. Hum. Commun. Res. 34, 287–318. 10.1111/j.1468-2958.2008.00322.x

[B9] Biocca F. Harms C. Burgoon J. K. (2003). Toward a more robust theory and measure of social presence: review and suggested criteria. Presence 12, 456–480. 10.1162/105474603322761270

[B10] Bowen J. Morosan C. (2018). Beware hospitality industry: the robots are coming. Worldw. Hosp. Tour. Themes. 10, 726–733. 10.1108/WHATT-07-2018-0045

[B11] Bowman D. Narayandas D. (2001). Managing customer-initiated contacts with manufacturers: the impact on share of category requirements and word-of-mouth behavior. J. Mark. Res. 38, 281–297. 10.1509/jmkr.38.3.281.18863

[B12] Brennan K. (2006). The managed teacher: Emotional labour, education, and technology. Educ. Insights. 10, 55–65.

[B13] Cho W. C. Lee K. Y. Yang S. B. (2019). What makes you feel attached to smartwatches? The stimulus-organism-response (S-O-R) perspectives. Inf. Technol. People 32, 319–343. 10.1108/ITP-05-2017-0152

[B14] Chung M. Ko E. Joung H. Kim S. J. (2020). Chatbot e-service and customer satisfaction regarding luxury brands. J. Bus. Res. 117, 587–595. 10.1016/j.jbusres.2018.10.004

[B15] Cronin J. J. Jr Taylor S. A. (1992). Measuring service quality: a reexamination and extension. J. Mark. 56, 55–68. 10.1177/002224299205600304

[B16] Cronin J. J. Jr Taylor S. A. (1994). SERVPERF versus SERVQUAL: reconciling performance-based and perceptions-minus-expectations measurement of service quality. J. Mark. 58, 125–131. 10.1177/002224299405800110

[B17] Dagger T. S. Sweeney J. C. Johnson L. W. (2007). A hierarchical model of health service quality: scale development and investigation of an integrated model. J. Serv. Res. 10, 123–142. 10.1177/1094670507309594

[B18] de Kervenoael R. Hasan R. Schwob A. Goh E. (2020). Leveraging human-robot interaction in hospitality services: incorporating the role of perceived value, empathy, and information sharing into visitors' intentions to use social robots. Tour. Manag. 78:104042. 10.1016/j.tourman.2019.104042

[B19] Dhingra S. Gupta S. Bhatt R. (2020). A study of relationship among service quality of e-commerce websites, customer satisfaction, and purchase intention. Int. J. E Bus. Res. 16, 42–59. 10.4018/IJEBR.2020070103

[B20] Emmers-Sommer T. M. (2004). The effect of communication quality and quantity indicators on intimacy and relational satisfaction. J. Soc. Pers. Relatsh. 21, 399–411. 10.1177/0265407504042839

[B21] Følstad A. Brandtzæg P. B. (2017). Chatbots and the new world of HCI. Interactions 24, 38–42. 10.1145/3085558

[B22] Fornell C. Larcker D. F. (1981). Structural equation models with unobservable variables and measurement error: algebra and statistics. J. Mark. Res. 18, 382–388. 10.1177/002224378101800313

[B23] George A. Kumar G. S. G. (2014). Impact of service quality dimensions in internet banking on customer satisfaction. Decision 41, 73–85. 10.1007/s40622-014-0028-2

[B24] Go E. Sundar S. S. (2019). Humanizing chatbots: the effects of visual, identity and conversational cues on humanness perceptions. Comput. Hum. Behav. 97, 304–316. 10.1016/j.chb.2019.01.020

[B25] Godey B. Manthiou A. Pederzoli D. Rokka J. Aiello G. Donvito R.. (2016). Social media marketing efforts of luxury brands: influence on brand equity and consumer behavior. J. Bus. Res. 69, 5833–5841. 10.1016/j.jbusres.2016.04.181

[B26] Grönroos C. (1984). A service quality model and its marketing implications. Eur. J. Mark. 18, 36–44. 10.1108/EUM0000000004784

[B27] Gummerus J. Liljander V. Pura M. Van Riel A. (2004). Customer loyalty to content-based web sites: the case of an online health-care service. J. Serv. Mark. 18, 175–186. 10.1108/08876040410536486

[B28] Gunawan D. Putri F. P. Meidia H. (2020). Bershca: bringing chatbot into hotel industry in Indonesia. Telkomnika 18, 839–845. 10.12928/telkomnika.v18i2.14841

[B29] Hair J. F. Black W. C. Babin B. J. Anderson R. E. Tatham R. L. (2006). Multivariate Data Analysis. Upper Saddle River, NJ: Pearson Prentice Hall.

[B30] Hair J. F. Hult G. T. M. Ringle C. Sarstedt M. (2013). A Primer on Partial Least Squares Structural Equation Modelling (PLS-SEM). London: Sage Publications.

[B31] Heeter C. (1989). “Implications of new interactive technologies for conceptualizing communication,” in Media Use in the Information Age: Emerging Patterns of Adoption and Computer Use, eds J. L. Salvaggio and J. Bryant (Hillsdale, NJ: Lawrence Erlbaum), 217–235.

[B32] Heung V. C. S. Cheng E. (2000). Assessing tourists' satisfaction with shopping in the Hong Kong Special Administrative Region of China. J. Travel. Res. 38, 396–404. 10.1177/004728750003800408

[B33] Holzwarth M. Janiszewski C. Neumann M. M. (2006). The influence of avatars on online consumer shopping behavior. J. Mark. 70, 19–36. 10.1509/jmkg.70.4.019

[B34] Huang M. H. Rust R. T. (2018). Artificial intelligence in service. J. Serv. Res. 21, 155–172. 10.1177/1094670517752459

[B35] Huo P. Yang Y. Zhou J. Chen C. He L. (2020). “TERG: topic-aware emotional response generation for chatbot,” in 2020 International Joint Conference on Neural Networks (IJCNN) (IEEE Publications), 1–8. 10.1109/IJCNN48605.2020.9206719

[B36] Janda S. Trocchia P. J. Gwinner K. P. (2002). Consumer perceptions of Internet retail service quality. Int. J. Serv. Ind. Manag. 13, 412–431. 10.1108/09564230210447913

[B37] Jang M. Jung Y. Kim S. (2021). Investigating managers' understanding of chatbots in the Korean financial industry. Comput. Hum. Behav. 120:106747. 10.1016/j.chb.2021.106747

[B38] Kang H. J. Kim S. I. (2017). Evaluation on the usability of chatbot intelligent messenger mobile services -Focusing on Google (Allo) and Facebook (M messenger). J. Korea Converg. Soc. 8, 271–276.

[B39] Kant R. Jaiswal D. (2017). The impact of perceived service quality dimensions on customer satisfaction: An empirical study on public sector banks in India. Int. J. Bank Mark. 35, 411–430. 10.1108/IJBM-04-2016-0051

[B40] Kassim N. Abdullah N. A. (2010). The effect of perceived service quality dimensions on customer satisfaction, trust, and loyalty in e-commerce settings: a cross cultural analysis. Asia Pac. J. Mark. Logist. 22, 351–371. 10.1108/13555851011062269

[B41] Kitapci O. Akdogan C. Dortyol I. T. (2014). The impact of service quality dimensions on patient satisfaction, repurchase intentions and word-of-mouth communication in the public healthcare industry. Proc. Soc. Behav. Sci. 148, 161–169. 10.1016/j.sbspro.2014.07.030

[B42] Kitapci O. Dortyol I. T. Yaman Z. Gulmez M. (2013). The paths from service quality dimensions to customer loyalty: an application on supermarket customers. Manag. Res. Rev. 36, 239–255. 10.1108/01409171311306391

[B43] Ladhari R. (2009). A review of twenty years of SERVQUAL research. Int. J. Qual. Serv. Sci. 1, 172–198. 10.1108/17566690910971445

[B44] Larivière B. Bowen D. Andreassen T. W. Kunz W. Sirianni N. J. Voss C.. (2017). “Service Encounter 2.0”: an investigation into the roles of technology, employees and customers. J. Bus. Res. 79, 238–246. 10.1016/j.jbusres.2017.03.008

[B45] Larivière B. Keiningham T. L. Aksoy L. Yalçin A. Morgeson F. V. I. I. I. Mithas S. (2016). Modeling heterogeneity in the satisfaction, loyalty intention, and shareholder value linkage: a cross-industry analysis at the customer and firm levels. J. Mark. Res. 53, 91–109. 10.1509/jmr.12.0143

[B46] Lee G. G. Lin H. F. (2005). Customer perceptions of e-service quality in online shopping. Int. J. Retail Distrib. Manag. 33, 161–176. 10.1108/09590550510581485

[B47] Lee H. Lee Y. Yoo D. (2000). The determinants of perceived service quality and its relationship with satisfaction. J. Serv. Mark. 14, 217–231. 10.1108/08876040010327220

[B48] Lee S. Choi J. (2017). Enhancing user experience with conversational agent for movie recommendation: effects of self-disclosure and reciprocity. Int. J. Hum. Comput. Stud. 103, 95–105. 10.1016/j.ijhcs.2017.02.005

[B49] Lee S. Comer L. B. Dubinsky A. J. Schafer K. (2011). The role of emotion in the relationship between customers and automobile salespeople. J. Manage. Issues 23, 206–226.

[B50] Lee Y. Kozar K. A. (2006). Investigating the effect of website quality on e-business success: an analytic hierarchy process (AHP) approach. Decis. Support Syst. 42, 1383–1401. 10.1016/j.dss.2005.11.005

[B51] Lehtinen U. Lehtinen J. R. (1991). Two approaches to service quality dimensions. Serv. Ind. J. 11, 287–303. 10.1080/02642069100000047

[B52] Lester J. Branting K. Mott B. (2004). “Conversational agents,” in The Practical Handbook of Internet Computing, ed M. P. Singh (Boca Raton, FL: Chapman and Hall; CRC Press), 220–240.

[B53] Li L. Lee K. Y. Emokpae E. Yang S. B. (2021). What makes you continuously use chatbot services? Evidence from Chinese online travel agencies. Electron. Mark. 31, 575–599. 10.1007/s12525-020-00454-z 35603227 PMC7817351

[B54] Liao C. Lin H. N. Luo M. M. Chea S. (2017). Factors influencing online shoppers' repurchase intentions: the roles of satisfaction and regret. Inf. Manag. 54, 651–668. 10.1016/j.im.2016.12.005

[B55] Liao Z. Cheung M. T. (2002). Internet-based e-banking and consumer attitudes: an empirical study. Inf. Manag. 39, 283–295. 10.1016/S0378-7206(01)00097-0

[B56] Lin Y. H. Lin K. Q. R. (2006). Assessing mainland Chinese visitors' satisfaction with shopping in Taiwan. Asia Pac. J. Tour. Res. 11, 247–268. 10.1080/10941660600753281

[B57] Liu B. Sundar S. S. (2018). Should machines express sympathy and empathy? Experiments with a health advice chatbot. Cyberpsychol. Behav. Soc. Netw. 21, 625–636. 10.1089/cyber.2018.0110 30334655

[B58] Liu F. Li J. Mizerski D. Soh H. (2012). Self-congruity, brand attitude, and brand loyalty: a study on luxury brands. Eur. J. Mark. 46, 922–937. 10.1108/03090561211230098

[B59] Long J. Yuan J. Lee H. M. (2019). How to program a chatbot-an introductory project and student perceptions. Issues Inform. Sci. Inf. Technol. 16, 1–31. 10.28945/4282

[B60] Luo X. Tong S. Fang Z. Qu Z. (2019). Frontiers: machines vs. humans: the impact of artificial intelligence chatbot disclosure on customer purchases. Mark. Sci. 38, 937–947. 10.1287/mksc.2019.1192

[B61] Madhavan P. Wiegmann D. A. Lacson F. C. (2006). Automation failures on tasks easily performed by operators undermine trust in automated aids. Hum. Factors 48, 241–256. 10.1518/001872006777724408 16884046

[B62] Markovic S. Iglesias O. Singh J. J. Sierra V. (2018). How does the perceived ethicality of corporate services brands influence loyalty and positive word-of-mouth? Analyzing the roles of empathy, affective commitment, and perceived quality. J. Bus. Ethics 148, 721–740. 10.1007/s10551-015-2985-6

[B63] Meilatinova N. (2021). Social commerce: factors affecting customer repurchase and word-of-mouth intentions. Int. J. Inf. Manag. 57:102300. 10.1016/j.ijinfomgt.2020.102300

[B64] Meyer-Waarden L. Pavone G. Poocharoentou T. Prayatsup P. Ratinaud M. Tison A.. (2020). How service quality influences customer acceptance and usage of chatbots? J. Serv. Manag. Res. 4, 35–51. 10.15358/2511-8676-2020-1-35

[B65] Mirnig N. Stollnberger G. Miksch M. Stadler S. Giuliani M. Tscheligi M. (2017). To err is robot: how humans assess and act toward an erroneous social robot. Front. Robot. AI 4:21. 10.3389/frobt.2017.00021

[B66] Moriuchi E. Landers V. M. Colton D. Hair N. (2021). Engagement with chatbots versus augmented reality interactive technology in e-commerce. J. Strat. Mark. 29, 375–389. 10.1080/0965254X.2020.1740766

[B67] Murray J. Elms J. Curran M. (2019). Examining empathy and responsiveness in a high-service context. Int. J. Retail. Distrib. Manag. 47, 1364–1378. 10.1108/IJRDM-01-2019-0016

[B68] Neuhofer B. Buhalis D. Ladkin A. (2015). Smart technologies for personalized experiences: a case study in the hospitality domain. Electron. Mark. 25, 243–254. 10.1007/s12525-015-0182-1

[B69] Nguyen C. Romaniuk J. (2014). Pass it on: a framework for classifying the content of word of mouth. Austral. Mark. J. 22, 117–124. 10.1016/j.ausmj.2013.12.014

[B70] Oliver R. L. (1980). A cognitive model of the antecedents and consequences of satisfaction decisions. J. Mark. Res. 17, 460–469. 10.1177/002224378001700405

[B71] Olorunniwo F. Hsu M. K. Udo G. J. (2006). Service quality, customer satisfaction, and behavioral intentions in the service factory. J. Serv. Mark. 20, 59–72. 10.1108/08876040610646581

[B72] Pan J.-N. Kuo T.-C. Bretholt A. (2010). Developing a new key performance index for measuring service quality. Ind. Manage. Data Syst. 110, 823–840. 10.1108/026355710110550729776949

[B73] Parasuraman A. Zeithaml V. A. Berry L. (1988). SERVQUAL: a multiple-item scale for measuring consumer perceptions of service quality. J. Retail. 64, 12–40.

[B74] Parasuraman A. Zeithaml V. A. Berry L. L. (1985). A conceptual model of service quality and its implications for future research. J. Mark. 49, 41–50. 10.1177/002224298504900403

[B75] Parasuraman A. Zeithaml V. A. Berry L. L. (1991). Refinement and reassessment of the SERVQUAL scale. J. Retail. 67, 420–450.

[B76] Parasuraman A. Zeithaml V. A. Malhotra A. (2005). E-S-QUAL: a multiple-item scale for assessing electronic service quality. J. Serv. Res. 7, 213–233. 10.1177/1094670504271156

[B77] Pillai R. Sivathanu B. (2020). Adoption of AI-based chatbots for hospitality and tourism. Int. J. Contemp. Hosp. Manage. 32, 3199–3226. 10.1108/IJCHM-04-2020-0259

[B78] Powell P. A. Roberts J. (2017). Situational determinants of cognitive, affective, and compassionate empathy in naturalistic digital interactions. Comput. Hum. Behav. 68, 137–148. 10.1016/j.chb.2016.11.024

[B79] Przegalinska A. Ciechanowski L. Stroz A. Gloor P. Mazurek G. (2019). In bot we trust: a new methodology of chatbot performance measures. Bus. Horiz. 62, 785–797. 10.1016/j.bushor.2019.08.005

[B80] Quah J. T. S. Chua Y. W. (2019). “Chatbot assisted marketing in financial service industry,” in Services Computing - SCC 2019. Lecture Notes in Computer Science, eds J. Ferreira, A. Musaev, and L. J. Zhang (Cham: Springer), 107–114. 10.1007/978-3-030-23554-3_8

[B81] Rese A. Ganster L. Baier D. (2020). Chatbots in retailers' customer communication: how to measure their acceptance? J. Retail. Consum. Serv. 56:102176. 10.1016/j.jretconser.2020.102176

[B82] Reynolds K. E. Beatty S. E. (1999). Customer benefits and company consequences of customer-salesperson relationships in retailing. J. Retail. 75, 11–32. 10.1016/S0022-4359(99)80002-5

[B83] Ribbink D. Van Riel A. C. R. Liljander V. Streukens S (2004). Comfort your online customer: quality, trust and loyalty on the internet. Manage. Serv. Qual. Int. J. 14, 446–456. 10.1108/09604520410569784

[B84] Roy R. Naidoo V. (2021). Enhancing chatbot effectiveness: the role of anthropomorphic conversational styles and time orientation. J. Bus. Res. 126, 23–34. 10.1016/j.jbusres.2020.12.051

[B85] Sands S. Ferraro C. Campbell C. Tsao H.-Y. (2020). Managing the human-chatbot divide: how service scripts influence service experience. J. Serv. Manage. 32, 246–264. 10.1108/JOSM-06-2019-0203

[B86] Seth N. Deshmukh S. G. Vrat P. (2005). Service quality models: a review. Int. J. Qual. Reliab. Manage. 22, 913–949. 10.1108/02656710510625211

[B87] Shah H. Warwick K. Vallverd,ú J. Wu D. (2016). Can machines talk? Comparison of Eliza with modern dialogue systems. Comput. Hum. Behav. 58, 278–295. 10.1016/j.chb.2016.01.004

[B88] Sharma N. (2018). Developing and validating an instrument for measuring online service quality in the tourism sector. IUP J. Manag. Res. 17, 38–70.

[B89] Shawar B. A. Atwell E. (2007). Chatbots: are they really useful? LDV Forum 22, 29–49.

[B90] Shawar B. A. Atwell E. S. (2005). Using corpora in machine-learning chatbot systems. Int. J. Corpus. Linguist. 10, 489–516. 10.1075/ijcl.10.4.06sha

[B91] Shin D. H. Hwang Y. Choo H. (2013). Smart TV: are they really smart in interacting with people? Understanding the interactivity of Korean Smart TV. Behav. Inf. Technol. 32, 156–172. 10.1080/0144929X.2011.603360

[B92] Shumanov M. Johnson L. (2021). Making conversations with chatbots more personalized. Comput. Hum. Behav. 117:106627. 10.1016/j.chb.2020.106627

[B93] Sivaramakrishnan S. Wan F. Tang Z. (2007). Giving an “e-human touch” to e-tailing: the moderating roles of static information quantity and consumption motive in the effectiveness of an anthropomorphic information agent. J. Interact. Mark. 21, 60–75. 10.1002/dir.20075

[B94] Tam J. L. M. (2004). Customer satisfaction, service quality and perceived value: an integrative model. J. Mark. Manage. 20, 897–917. 10.1362/0267257041838719

[B95] Thomas P. Czerwinski M. McDuff D. Craswell N. Mark G. (2018). “Style and alignment in information-seeking conversation,” in Proceedings of the 2018 Conference on Human Information Interaction and Retrieval (CHIIR 2018) (New York, NY: Association for Computing Machinery), 42–51. 10.1145/3176349.3176388

[B96] Tussyadiah I. (2020). A review of research into automation in tourism: launching the annals of tourism research curated collection on artificial intelligence and robotics in tourism. Ann. Tour. Res. 81:102883. 10.1016/j.annals.2020.102883

[B97] Van Doorn J. Mende M. Noble S. M. Hulland J. Ostrom A. L. (2017). Domo arigato Mr. Roboto: emergence of automated social presence in organizational frontlines and customers' service experiences. J. Serv. Res. 20, 43–58. 10.1177/1094670516679272

[B98] Verkijika S. F. De Wet L. (2019). Understanding word-of-mouth (WOM) intentions of mobile app users: the role of simplicity and emotions during the first interaction. Telemat. Inform. 41, 218–228. 10.1016/j.tele.2019.05.003

[B99] Wexelblat A. (1998). Don't make that face: a report on anthropomorphizing an interface. Intell. Environ, 173–179.

[B100] Winkler R. Soellner M. (2018). “Unleashing the potential of chatbots in education: a state-of-the-art analysis,” in Academy of Management Annual Meeting (AOM). (Chicago), 15903. 10.5465/AMBPP.2018.15903abstract

[B101] Wirtz J. Patterson P. G. Kunz W. H. Gruber T. Lu V. N. Paluch S.. (2018). Brave new world: service robots in the frontline. J. Serv. Manage. 29, 907–931. 10.1108/JOSM-04-2018-0119

[B102] Wolfinbarger M. Gilly M. C. (2003). eTailQ: dimensionalizing, measuring and predicting etail quality. J. Retail. 79, 183–198. 10.1016/S0022-4359(03)00034-4

[B103] Yin J. Goh T. T. Yang B. Xiaobin Y. (2021). Conversation technology with micro-learning: the impact of chatbot-based learning on students' learning motivation and performance. J. Educ. Comput. Res. 59, 154–177. 10.1177/0735633120952067

[B104] Yoo W. S. Lee Y. Park J. (2010). The role of interactivity in e-tailing: creating value and increasing satisfaction. J. Retail. Cons. Serv. 17, 89–96. 10.1016/j.jretconser.2009.10.003

[B105] Zeithaml V. A. (2002). Service excellence in electronic channels. Manage. Serv. Qual. Int. J. 12, 135–139. 10.1108/09604520210429187

[B106] Zeithaml V. A. Berry L. L. Parasuraman A. (1996). The behavioral consequences of service quality. J. Mark. 60, 31–46. 10.1177/002224299606000203

[B107] Zeithaml V. A. Parasuraman A. Berry L. L. (1990). Delivering Quality Service: Balancing Customer Perceptions and Expectations. New York, NY: Simon and Schuster.

[B108] Zhang J. Oh Y. J. Lange P. Yu Z. Fukuoka Y. (2020). Artificial intelligence Chatbot behavior change model for designing artificial intelligence Chatbots to promote physical activity and a healthy diet: viewpoint. J. Med. Intern. Res. 22:e22845. 10.2196/22845 32996892 PMC7557439

[B109] Zhang W. N. Zhu Q. Wang Y. Zhao Y. Liu T. (2019). Neural personalized response generation as domain adaptation. World Wide Web. 22, 1427–1446. 10.1007/s11280-018-0598-629665538

[B110] Zhu F. X. Wymer W. Chen I. (2002). IT-based services and service quality in consumer banking. Int. J. Serv. Ind. Manage. 13, 69–90. 10.1108/09564230210421164

