# Supplementary material for: The Effects of Chatbot Service Recovery With Emotion Words on Customer Satisfaction, Repurchase Intention, and Positive Word-Of-Mouth
Source: Front Psychol. 2022 May 31;13:922503. doi: 10.3389/fpsyg.2022.922503 (PMC9194808; doi:10.3389/fpsyg.2022.922503)
Supplement: Supplementary file 1 [file Data_Sheet_1.docx]

Supplementary Material

# Supplementary Tables

# Supplementary Table 1 Emotion label

| **Emotion Label** | Message: My favorite sport is basketball. | |
| --- | --- | --- |
|  | Generated responses | Real-life responses |
| **Happy** | What a happy day! | I will be very excited after a game of basketball. |
| **Like** | I like it. | I also like playing basketball. |
| **Disgust** | I hate playing piano. | I find basketball too difficult. |
| **Sad** | I am so sad. | The team lost yesterday, and the players were frustrated. |
| **Angry** | I am so angry. | I am very annoyed because I cannot throw the ball into the basket. |
| **Null** | We will not play basketball tomorrow. | No one doubts Kobe’s basketball skills. |

**Supplementary Table 2 Measurement items**

| **Construct** | **Measurement items** | **Sources** |
| --- | --- | --- |
| Reliability | 1. This chatbot is dependable.  2. When I have problems, this chatbot is sympathetic and reassuring.  3. I felt that I could rely on the chatbot’s services to fulfill my needs. | Parasuraman et al. (1988) and Li et al. (2021) |
| Responsiveness | 1. This chatbot provides prompt services that meet my expectations.  2. This chatbot responds promptly to my requests.  3. This chatbot provides services exactly when I need them without any delay. | Parasuraman et al. (1988) and Li et al. (2021) |
| Assurance | 1. I trust this chatbot.  2. I feel safe and assured to have a conversation with this chatbot.  3. This chatbot has adequate knowledge to answer my questions. | Parasuraman et al. (1988) and Li et al. (2021) |
| Interactivity | 1. I can be in control of my personal needs through this chatbot.  2. This chatbot is sensitive to my personal needs  3. This chatbot gives me the opportunity to respond. | Li et al. (2021) |
| Empathy | 1. I feel valued by this brand through my conversations with this chatbot.  2. I feel empathetically understood through the conversation with this chatbot.  3. I feel that this chatbot was developed to meet my personal needs. | Parasuraman et al. (1988) |
| Customer satisfaction | 1. This chatbot did a good job.  2. This chatbot did what I expected.  3. Overall, I am satisfied with my experience of using this chatbot. | Parasuraman et al. (1988) and Li et al. (2021) |
| Repurchase intention:  After interacting with this chatbot, the likelihood of me buying products from this brand in the future is | 1. Very improbable . . . very probable  2. Impossible . . . possible  3. No chance . . . certain | Moriuchi et al. (2021) |
| Positive word-of-mouth | 1. I intend to recommend this brand to others after using this chatbot.  2. I intend to speak of this brand’s positive side after using this chatbot.  3. I intend to strongly recommend people to avail services from this brand’s chatbot. | Bagherzadeh et al. (2020) |
